# Integrated Bulk and Single‐Cell Transcriptomic Analyses Identify a Five‐Gene Signature Associated With Prognosis and the Tumor Microenvironment in Epstein–Barr Virus–Associated Gastric Cancer

**DOI:** 10.1155/genr/5991107

**Published:** 2026-08-29

**Authors:** Lufei Jin, Man Jiang, Yubin Pan, Yan Wang, Lei Zhang, Wujie Chen

**Affiliations:** ^1^ Department of Radiology, Zhejiang Cancer Hospital, Hangzhou Institute of Medicine (HIM), Chinese Academy of Sciences, Hangzhou Zhejiang, 310022, China, cas.cn

**Keywords:** biomarkers, EBV-associated gastric cancer, single-cell RNA sequencing, transcriptomics, tumor microenvironment

## Abstract

**Background:**

Epstein–Barr virus–associated gastric cancer (EBVaGC) is a distinct molecular subtype of gastric cancer, but reliable biomarkers linking EBV‐related biology, prognosis, and microenvironmental remodeling remain limited.

**Methods:**

Differentially expressed genes associated with EBVaGC were identified from TCGA and GEO datasets. Weighted gene co‐expression network analysis and machine learning were used to screen core genes. Functional enrichment, diagnostic and prognostic modeling, immune infiltration analysis, single‐cell transcriptomic analysis of a public scRNA‐seq dataset, cell–cell communication analysis, drug sensitivity prediction, external cohort validation, and RT‐qPCR validation in gastric cancer cell lines were subsequently performed.

**Results:**

Five core genes (ASPA, CHODL, GNG7, P2RY14, and PI16) were identified. The combined classifier showed high apparent diagnostic performance in the discovery datasets (AUC = 1.000 in the EBV cohort and 0.981 in TCGA–STAD) and retained value in GSE27342 (AUC = 0.771). RT‐qPCR confirmed differential expression of these genes between a gastric epithelial cell line and a gastric cancer cell line, providing general tumor‐versus‐normal experimental support rather than EBV‐specific validation. A five‐gene risk signature stratified overall survival, with 1‐, 2‐, and 3‐year AUCs of 0.634, 0.645, and 0.622, which improved to 0.716, 0.768, and 0.693 after integration with age and stage. Single‐cell analysis localized the strongest signature signal to fibroblasts and B cells and revealed enhanced MIF‐, PTN‐, and TNFSF13B‐related communication in tumors. High‐risk tumors showed higher predicted IC50 values for paclitaxel and 5‐fluorouracil.

**Conclusion:**

We identified a five‐gene signature with strong diagnostic value and biologically meaningful prognostic relevance in EBVaGC, highlighting fibroblast‐ and B‐cell‐associated microenvironmental programs in aggressive disease.

## 1. Introduction

Gastric cancer (GC) remains a major global health challenge, ranking among the most common malignancies and the leading causes of cancer‐related death worldwide [1]. Despite advances in surgery, chemotherapy, targeted therapy, and immunotherapy, the clinical management of GC remains difficult because of its marked molecular and clinical heterogeneity [2]. Patients with apparently similar clinicopathological characteristics often experience substantially different outcomes and treatment responses, highlighting the limitations of conventional staging systems in accurately capturing the biological complexity of the disease [3]. To address this issue, molecular classification has become an important framework for refining GC stratification. The Cancer Genome Atlas (TCGA) classified GC into four molecular subtypes, namely Epstein–Barr virus (EBV)–positive, microsatellite instability (MSI), genomically stable (GS), and chromosomal instability (CIN), providing a new basis for understanding the heterogeneity of GC at the molecular level [4].

Among these subtypes, Epstein‐Barr virus–associated gastric cancer (EBVaGC) has been recognized as a distinct clinicopathological entity. EBVaGC is characterized by the monoclonal presence of the EBV genome within malignant epithelial cells and accounts for approximately 8%–10% of all GC [5, 6]. Compared with other GC subtypes, EBVaGC typically exhibits unique histological and molecular features, including lymphoepithelioma‐like morphology, abundant immune‐cell infiltration, and extensive epigenetic alterations such as widespread CpG island hypermethylation [7–9]. These characteristics indicate that EBVaGC is not merely a histological variant of GC but a biologically distinct subtype driven by virus–host interactions, altered immune regulation, and epigenetic remodeling.

Accumulating evidence has further demonstrated that EBV infection plays an important role in gastric carcinogenesis and in shaping the tumor microenvironment of EBVaGC. Previous transcriptomic, epigenetic, and immunological studies have shown that EBVaGC displays distinct gene expression patterns, immune activation features, and stromal–immune interactions compared with EBV‐negative GC [10–12]. In particular, the immune‐rich nature of EBVaGC has attracted increasing attention, as it suggests that viral infection may influence not only malignant epithelial cells but also the surrounding fibroblast, B‐cell, and other immune‐cell compartments [13, 14]. With the rapid development of high‐throughput sequencing technologies, bulk transcriptomic profiling has enabled the identification of disease‐associated genes and molecular programs in large cohorts, whereas single‐cell RNA (scRNA) sequencing has provided a higher‐resolution view of the cellular composition and intercellular communication within the tumor microenvironment. Together, these studies have greatly expanded our understanding of the biological characteristics of EBVaGC.

However, although numerous candidate genes have been reported in GC, biomarkers that are simultaneously useful for diagnosis, informative for prognosis, and mechanistically linked to the tumor microenvironment remain limited in EBVaGC. Moreover, the cellular context underlying these biomarkers has not been fully clarified, especially with respect to stromal and immune components such as fibroblasts and B cells. Therefore, identifying key molecular markers that are closely associated with EBVaGC and characterizing their microenvironmental context are necessary for improving biological understanding and for developing more precise diagnostic and prognostic strategies.

Based on these considerations, the present study aimed to identify EBVaGC‐related core genes through integrative analysis of public transcriptomic datasets and to further evaluate their diagnostic value, prognostic significance, and tumor microenvironmental relevance. In addition, by integrating bulk transcriptomic analysis with a public single‐cell transcriptomic dataset, this study sought to clarify the cellular distribution and intercellular communication features associated with these candidate biomarkers, thereby providing a more comprehensive understanding of their biological and potential clinical significance in EBVaGC.

## 2. Methods

### 2.1. Data Acquisition and Preprocessing

Transcriptomic and clinical data for GC were retrieved from The TCGA and Gene Expression Omnibus (GEO) databases. TCGA–STAD RNA‐seq data and corresponding clinical information were used as the main bulk transcriptomic cohort. GSE51575 was used to identify EBV‐related transcriptional alterations, and GSE27342 was used for external assessment of tumor‐versus‐normal diagnostic relevance. In addition, the publicly available GC scRNA‐seq dataset GSE275648 was obtained from GEO and used to characterize the cellular distribution of the five‐gene signature and the associated tumor microenvironmental context.

### 2.2. Identification of EBV‐Related Differentially Expressed Genes (DEGs)

Differential expression analysis was performed using the limma package. For TCGA–STAD, DEGs between tumor and normal tissues were identified. For GSE51575, DEGs were screened between EBV‐positive and EBV‐negative tumor samples. Significance thresholds were set at |log_2FC| > 1 and adj.P.Val < 0.05. The intersection of upregulated and downregulated genes across datasets was identified and visualized using Venn diagrams. The two discovery contrasts were intentionally complementary: GSE51575 captured EBV‐associated differences among tumors, whereas TCGA–STAD captured tumor‐associated differences relative to normal tissue. Accordingly, the intersected genes were interpreted as EBV‐associated and tumor‐relevant candidates rather than as biomarkers validated for a single classification task.

### 2.3. Functional Enrichment Analysis

To explore the biological roles of the intersection genes, Gene Ontology (GO) and Kyoto Encyclopedia of Genes and Genomes (KEGG) enrichment analyses were conducted using the clusterProfiler R package. *p* values were adjusted using the Benjamini–Hochberg (BH) method, with a significance cutoff of 0.05.

### 2.4. Weighted Gene Co‐Expression Network Analysis (WGCNA)

To identify gene modules most relevant to EBV infection and tumor progression, we constructed co‐expression networks using the WGCNA package. First, genes were filtered based on the median absolute deviation (MAD), retaining the top 25% most variable genes. A sample clustering analysis was performed to detect and remove outliers. We then utilized the PickSoftThreshold function to calculate the scale‐free topology fit index and select the optimal soft‐thresholding power (β). After establishing the adjacency matrix, it was transformed into a topological overlap matrix (TOM) to evaluate the relative interconnectedness of gene pairs. We employed a dynamic tree‐cutting algorithm to identify distinct modules. Finally, Module Eigengenes (ME) were correlated with clinical traits (EBV status and tumor status) using Spearman correlation to select key modules for downstream feature extraction.

### 2.5. Machine Learning‐Based Feature Selection

To refine the candidate gene pool, two robust machine learning algorithms were integrated. First, the random forest (RF) algorithm was applied with 500 trees, ranking features by mean decrease in Gini. Second, support vector machine–recursive feature elimination (SVM—RFE) was implemented with 5‐fold cross‐validation to identify the feature subset with the highest diagnostic accuracy. Genes consistently identified across two models in both GEO and TCGA cohorts were designated as core biomarkers. In addition, machine learning–based feature selection (LASSO and SVM–RFE) was performed on the full discovery datasets without splitting them into training and test sets, as the primary aim was to identify robust candidate genes rather than to construct a predictive model at this stage.

### 2.6. Diagnostic and Prognostic Model Construction

Diagnostic efficacy was quantified using receiver operating characteristic (ROC) curve analysis via the pROC package, calculating the area under the curve (AUC) for both individual biomarkers and multigene classifiers. For survival analysis, TCGA–STAD patients with complete clinical follow‐up were included. Univariate and multivariate Cox proportional hazards regression analyses were conducted to identify independent prognostic factors and calculate hazard ratios (HR). A prognostic risk score was calculated using the following formula:

(1) Risk score=∑i=1nCoefi×Expi,

where Coef_*i*_ represents the multivariate Cox regression coefficient and Exp_*i*_ denotes the expression level of gene i. Patients were stratified into high‐ and low‐risk groups based on the optimal cutoff determined by the surv_cutpoint function. Survival differences were visualized using Kaplan–Meier (KM) curves and assessed via the log‐rank test. Time‐dependent ROC curves were generated using the timeROC package to evaluate the 1‐, 2‐, and 3‐year predictive accuracy of the model.

To benchmark prognostic performance, TNM stage‐only, age‐plus‐stage, five‐gene, and combined clinical‐genomic Cox models were fitted in the same complete‐case cohort. Incremental genomic value was evaluated using likelihood‐ratio tests, with bootstrap estimation of C‐index and time‐dependent AUC. Because CHODL showed nonproportional hazards, a sensitivity model allowing its coefficient to vary with log time was additionally evaluated.

### 2.7. Immune Infiltration Analysis

Immune infiltration levels were quantified using single‐sample Gene Set Enrichment Analysis (ssGSEA) with a set of 28 immune cell markers. Additionally, MCPcounter and ESTIMATE algorithms were utilized to estimate the abundance of immune and stromal cells. Correlations between biomarker expression, risk scores, and immune cell abundance were analyzed using Spearman correlation.

### 2.8. scRNA Analysis

A publicly available GC scRNA‐seq dataset was reanalyzed using the Seurat (v5) package to characterize the cellular distribution of the five‐gene signature and the associated tumor microenvironment. Quality control (QC) involved filtering cells with nFeature_RNA < 200 or > 4000 and excluding cells with high mitochondrial content. After log normalization and selection of 3000 highly variable genes, PCA was performed. Harmony correction used sample ID as the batch variable. The first 30 Harmony dimensions were used for neighbor construction, Louvain clustering (resolution = 0.5), and t‐SNE.

Cell‐type annotation was performed using SingleR with the Human Primary Cell Atlas (HPCA) reference and verified with canonical markers. Risk signature scores were calculated at the single‐cell level using the AddModuleScore function. Intercellular communication was inferred using the CellChat package, utilizing the Secreted Signaling database and the triMean communication probability method to compare signaling strengths and network centrality (in‐degree and out‐degree) between tumor and normal states.

### 2.9. Drug Sensitivity Prediction

The oncoPredict package was used to estimate the half‐maximal inhibitory concentration (IC50) of common chemotherapy drugs based on the GDSC database.

### 2.10. Cell Culture

Reverse transcription‐quantitative PCR (RT‐qPCR) validation was performed using the human normal gastric epithelial cell line GES‐1 and the human GC cell line HGC‐27. GES‐1 is a human SV40‐transformed gastric epithelial cell line derived from fetal stomach epithelium; the donor sex is not specified in the available cell line records. HGC‐27 is a human gastric carcinoma cell line derived from a metastatic lymph node of GC; the donor sex is not specified in the available cell line records. Both cell lines were obtained from Biovector NTCC Cell Culture Collection, Inc. (Beijing, China) in January 2026. Cells were cultured in RPMI‐1640 medium (Gibco, New York, NY, USA) supplemented with 10% fetal bovine serum and 1% penicillin–streptomycin at 37°C in a humidified incubator containing 5% CO_2_. The GES‐1 and HGC‐27 cells used in the RT‐qPCR validation experiments described in this study were authenticated by short tandem repeat (STR) profiling. STR profiles were compared with reference profiles in DSMZ, with matching rates of 100% for GES‐1 and HGC‐27. Mycoplasma contamination was assessed using mycoplasma detection PCR, and both cell lines tested negative before RNA extraction.

### 2.11. RT‐qPCR Analysis

When cells reached appropriate confluence, total RNA was extracted using TRIzol reagent (Tiangen, Beijing, China) according to the manufacturer’s instructions. RNA concentration and purity were determined using a spectrophotometer, and only qualified RNA samples were used for subsequent analyses.

Then, complementary DNA (cDNA) was synthesized from total RNA using the PrimeScript RT Reagent Kit (Takara, Japan) in accordance with the manufacturer’s protocol. Quantitative PCR was carried out on an ABI 7500 Real‐Time PCR System using SYBR Green PCR Master Mix (RR066A; Takara, Kameoka, Japan). The PCR amplification protocol consisted of an initial denaturation at 95°C for 2–3 min, followed by 40 cycles of denaturation at 95°C for 10–15 s and annealing/extension at 60°C for 30–60 s. A melt curve analysis was performed at the end of the amplification to verify product specificity. Each sample was analyzed in quadruplicate, and at least four independent biological replicates were included.

The relative mRNA expression levels of ASPA, CHODL, GNG7, P2RY14, and PI16 were normalized to the internal control GAPDH, and expression differences between GES‐1 and HGC‐27 cells were calculated using the comparative Ct method (2^−ΔΔCt^). Primer sequences are listed in Table S1.

### 2.12. Statistical Analysis

All statistical analyses were performed in R software. Differential expression analysis was conducted using the limma package, with adjusted *p* values calculated using the BH method where applicable. Spearman correlation analysis was used to evaluate associations between biomarker expression, risk scores, and immune infiltration levels. KM survival analysis was performed and compared using the log‐rank test, while univariate and multivariate Cox proportional hazards regression models were used to determine prognostic significance. Differences in estimated drug sensitivity between two groups were assessed using the Wilcoxon rank‐sum test. Diagnostic and prognostic performance were evaluated by ROC curve analysis. Unless otherwise indicated, all tests were two‐sided, and *p* < 0.05 was considered statistically significant.

## 3. Results

### 3.1. Identification of Common DEGs Associated With EBV and Tumor Status

**FIGURE 1:**
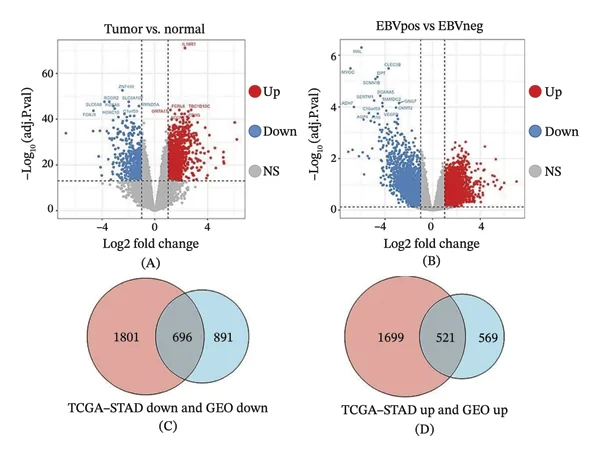
Identification of common differentially expressed genes (DEGs) associated with EBV infection and gastric tumor status. (A) Volcano plot showing DEGs between tumor and normal tissues in the TCGA–STAD cohort. (B) Volcano plot showing DEGs between EBV‐positive and EBV‐negative samples in the GSE51575 cohort. (C) Venn diagram of the overlapping downregulated genes shared by the TCGA–STAD and GSE51575 datasets. (D) Venn diagram of the overlapping upregulated genes shared by the TCGA–STAD and GSE51575 datasets.

To identify key drivers in EBVaGC, we initiated differential expression analysis across two cohorts. In the TCGA–STAD dataset, a total of 2497 downregulated and 2220 upregulated genes were identified in tumor tissues compared to normal controls (Figure 1A). In the GSE51575 cohort, we identified 1587 downregulated and 1090 upregulated genes associated with EBV‐positive status (Figure 1B). By intersecting these results, we identified 696 common downregulated genes and 521 common upregulated genes shared between the TCGA and GEO datasets (Figure 1C,D).

### 3.2. Functional Enrichment of Intersection Genes

**FIGURE 2:**
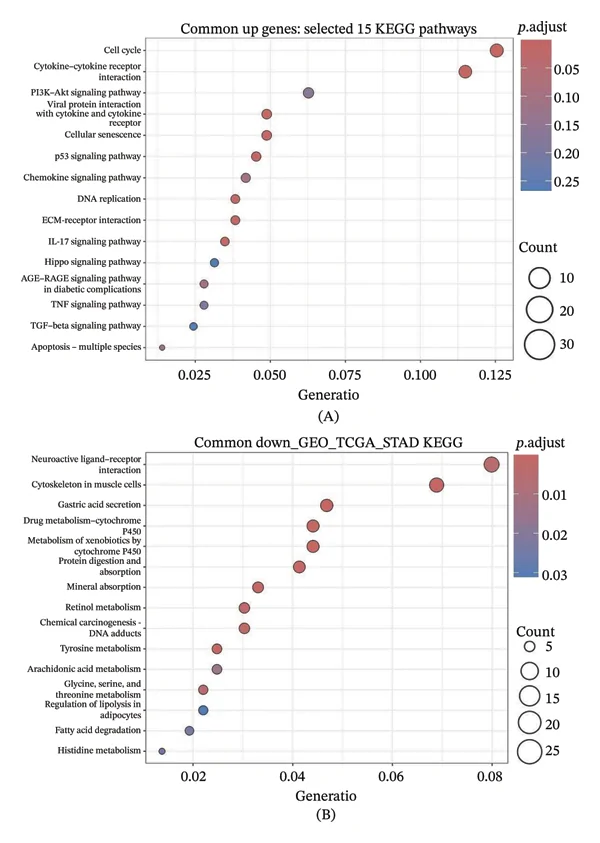
KEGG enrichment analysis of overlapping upregulated and downregulated genes. (A) Bubble plot showing the top enriched KEGG pathways for the common upregulated genes shared between TCGA–STAD and GSE51575. (B) Bubble plot showing the top enriched KEGG pathways for the common downregulated genes.

To elucidate the biological processes involved, we performed KEGG enrichment analysis on the intersection genes. The common upregulated genes were significantly enriched in pathways related to the cell cycle, cytokine–cytokine receptor interaction, and the PI3K–Akt signaling pathway (Figure 2A). Conversely, common downregulated genes were predominantly associated with metabolic pathways, such as retinol metabolism and tyrosine metabolism (Figure 2B).

### 3.3. Identification of Key Modules via WGCNA

**FIGURE 3:**
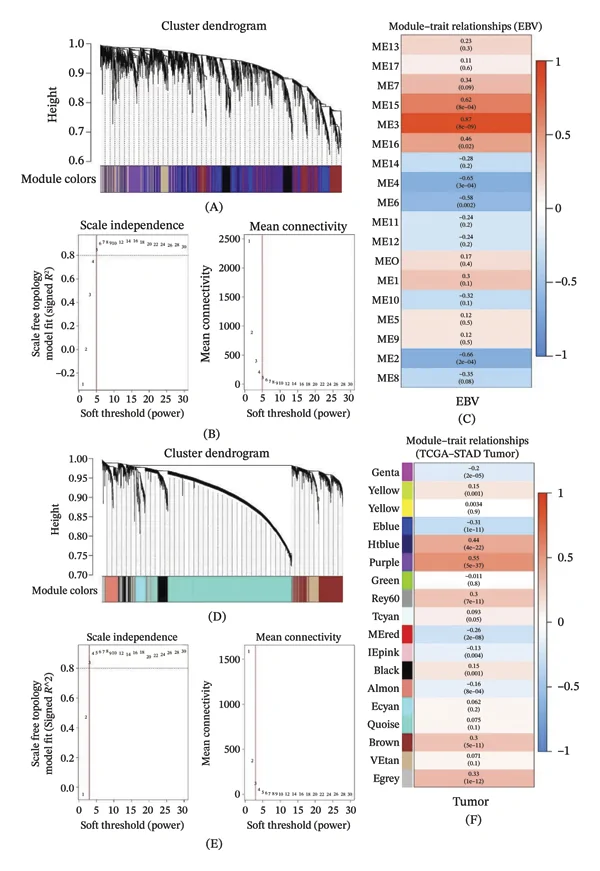
WGCNA identifies EBV‐ and tumor‐associated co‐expression modules. (A) Gene dendrogram and module assignment in the GSE51575 cohort. (B) Scale‐free topology fit index and mean connectivity across a series of soft‐thresholding powers for the GSE51575 dataset. (C) Heatmap of module–trait relationships between gene modules and EBV infection status in GSE51575. (D) Gene dendrogram and module assignment in the TCGA–STAD cohort. (E) Scale‐free topology fit index and mean connectivity across candidate soft‐thresholding powers for the TCGA–STAD dataset. (F) Heatmap of module–trait relationships between gene modules and tumor status in TCGA–STAD.

To further narrow down the gene set to those most biologically relevant, we performed WGCNA to identify key co‐expression modules. In the GEO dataset, we selected a soft‐thresholding power of *β* = 5 to satisfy scale‐free topology (Figure 3A,B). Among the identified modules, the ME3 module showed the strongest positive correlation with EBV infection (*R* = 0.87, *p* < 0.01), whereas the ME2 module exhibited a strong negative correlation (Figure 3C). Parallel analysis in the TCGA cohort utilized a power of *β* = 3 (Figure 3E). We found that several modules, notably the purple module ($*R* = 0.55, *p* < 0.01), were significantly associated with tumor status (Figure 3F). The genes belonging to these high‐correlation modules were intersected with the previously identified common DEGs to ensure both differential expression and network‐level significance (Figure S1).

### 3.4. Hub Gene Selection Through Machine Learning

**FIGURE 4:**
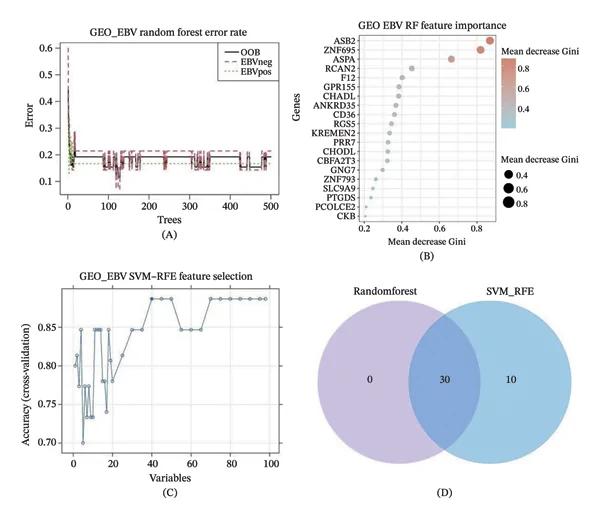
Machine‐learning–based feature selection in the GEO EBV cohort. (A) Random forest error rate across increasing numbers of trees in the GSE51575 dataset. (B) Variable importance ranking of candidate genes identified by random forest, measured by mean decrease in Gini index. (C) Support vector machine–recursive feature elimination (SVM–RFE) curve showing cross‐validation accuracy across different numbers of retained variables. (D) Venn diagram showing the overlap between genes selected by random forest and SVM–RFE in the GEO EBV cohort.

**FIGURE 5:**
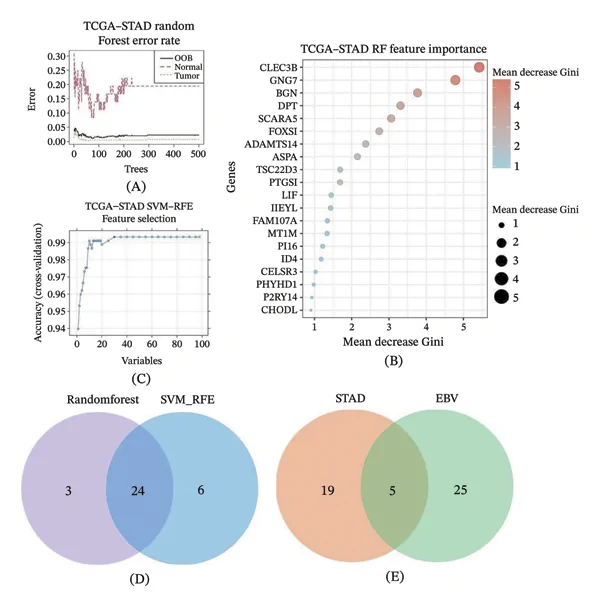
Machine‐learning–based feature selection in the TCGA–STAD cohort and identification of shared core biomarkers. (A) Random forest error rate across increasing tree numbers in the TCGA–STAD dataset. (B) Variable importance ranking of candidate genes identified by random forest in TCGA–STAD. (C) SVM–RFE curve showing cross‐validation accuracy for feature selection in TCGA–STAD. (D) Venn diagram showing the overlap between genes selected by random forest and SVM–RFE in the TCGA–STAD cohort. (E) Venn diagram showing the intersection between the feature genes obtained from the TCGA–STAD and GEO EBV cohorts.

To refine the gene set, we applied multiple machine learning models. For the GEO_EBV dataset, RF and SVM–RFE identified 30 overlapping genes as core features (Figure 4). In the TCGA_STAD dataset, 24 genes were consistently selected by both models (Figure 5D). Intersection of the feature genes from both cohorts ultimately yielded 5 core biomarkers: ASPA, CHODL, GNG7, P2RY14, and PI16 (Figure 5E).

### 3.5. Diagnostic Efficacy of Core Biomarkers

**FIGURE 6:**
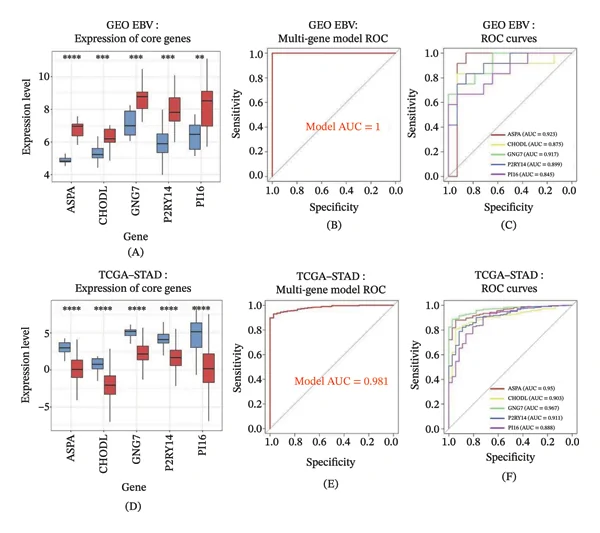
In‐sample diagnostic performance in the discovery cohorts of the five‐gene signature. (A) Expression levels of ASPA, CHODL, GNG7, P2RY14, and PI16 in the GEO EBV cohort. Blue indicated an EBV‐negative sample, and red indicated EBV‐positive samples. (B) Receiver operating characteristic (ROC) curve of the combined five‐gene model in the GEO EBV cohort. (C) ROC curves of the individual biomarkers in the GEO EBV cohort. (D) Expression levels of the five core biomarkers in the TCGA–STAD cohort. Blue indicated normal sample, and red indicated tumor samples. (E) ROC curve of the combined five‐gene model in the TCGA–STAD cohort. (F) ROC curves of the individual biomarkers in the TCGA–STAD cohort.

The expression and diagnostic performance of the five core biomarkers were first evaluated in the internal discovery cohorts. In both EBV and TCGA–STAD, all five genes were significantly differentially expressed between groups, consistent with the previous results (Figure 6A,D). The combined multigene classifier showed excellent discrimination, with an AUC of 1.000 in the GEO_EBV cohort and 0.981 in the TCGA–STAD cohort (Figure 6B,E). Individual biomarkers also demonstrated strong diagnostic performance. In GEO_EBV, the AUCs were 0.923, 0.875, 0.917, 0.899, and 0.845, respectively (Figure 6C), while the corresponding AUCs were 0.950, 0.903, 0.967, 0.911, and 0.888 in TCGA–STAD, respectively (Figure 6F).

### 3.6. External Validation of Diagnostic Value

**FIGURE 7:**
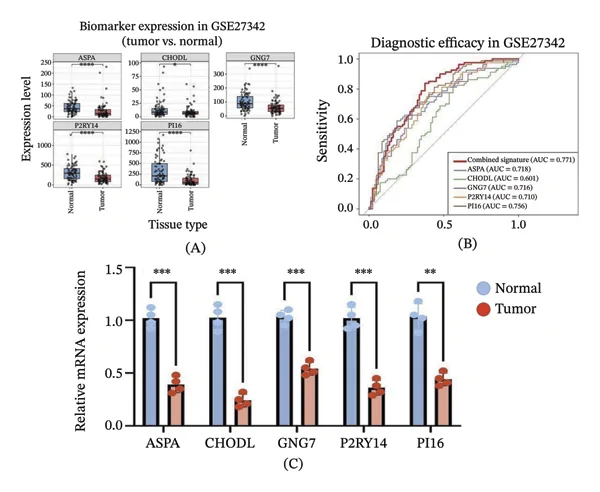
External validation of the diagnostic value of the five‐gene signature. (A) Expression levels of the five core biomarkers in the external GSE27342 cohort. (B) ROC curve of the combined five‐gene model for discriminating tumor from normal samples in GSE27342. (C) Relative expression of ASPA, CHODL, GNG7, P2RY14, and PI16 determined by RT‐qPCR in GES‐1 and HGC‐27 cells.

To further evaluate the robustness of the five‐gene signature, we performed validation analyses in independent datasets at both the expression and diagnostic levels. In GSE27342, all five core biomarkers remained significantly differentially expressed between tumor and normal tissues, and their expression patterns were consistent with those observed in the TCGA cohorts (Figure 7A). Besides, the combined five‐gene classifier retained diagnostic value, with an AUC of 0.771 in discriminating tumor from normal samples (Figure 7B). Because EBV status was not available in the metadata used for GSE27342, this external validation assessed tumor‐versus‐normal discrimination rather than EBV‐positive versus EBV‐negative discrimination.

Then, we examined their expression in GES‐1 and HGC‐27 cells using RT‐qPCR. The results showed that ASPA, CHODL, GNG7, P2RY14, and PI16 were significantly dysregulated in HGC‐27 cells relative to GES‐1 cells, and the overall expression trends were consistent with the transcriptomic findings (Figure 7C). These RT‐qPCR findings provide general cell‐line‐level support for expression differences between a gastric epithelial line and a GC line; they do not establish EBV specificity or functional roles for the five genes.

### 3.7. Prognostic Significance and Risk Model Construction

**FIGURE 8:**
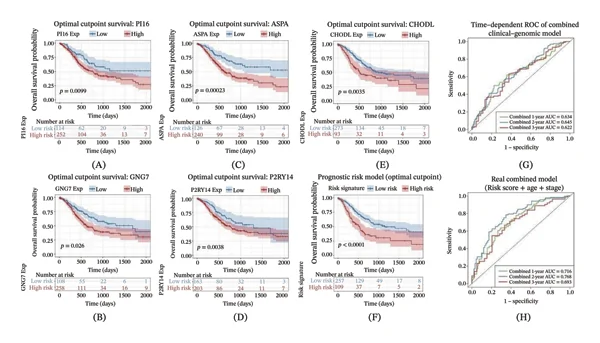
Prognostic significance of the five biomarkers and construction of the risk model. (A–E) Kaplan–Meier survival curves showing the prognostic impact of PI16, ASPA, CHODL, GNG7, and P2RY14 expression in the TCGA–STAD cohort. (F) Kaplan–Meier survival curve comparing overall survival between the high‐risk and low‐risk groups in the TCGA–STAD cohort. (G) Time‐dependent ROC curves evaluating the predictive performance of the multivariate risk model in TCGA–STAD. (H) Time‐dependent ROC curves evaluating the predictive performance of the combined clinical‐genomic model integrating risk score, age, and stage in the TCGA–STAD cohort.

To assess prognostic relevance, KM survival analysis was performed in the TCGA–STAD cohort. High expression of PI16, GNG7, ASPA, P2RY14, and CHODL was significantly associated with shorter overall survival (OS) (Figure 8A–E). A multivariable Cox model was then used to construct a risk signature, which stratified patients into high‐ and low‐risk groups.

Patients in the high‐risk group had significantly worse OS than those in the low‐risk group (*p* < 0.0001; Figure 8F). Time‐dependent ROC analysis of the genomic model showed moderate predictive performance, with AUCs of 0.634, 0.645, and 0.622 at 1, 2, and 3 years, respectively (Figure 8G). After integrating risk score with age and stage, the combined clinical‐genomic model improved prognostic discrimination, with AUCs of 0.716, 0.768, and 0.693 at 1, 2, and 3 years, respectively (Figure 8H).

In 314 patients with 111 deaths, the apparent C‐indices were 0.628 for TNM stage, 0.663 for age plus stage, 0.607 for the five‐gene model, and 0.694 for the combined model (Table S2). Although the combined model showed numerically higher discrimination, the conventional likelihood‐ratio test did not reach statistical significance (*p* = 0.082). A sensitivity analysis accounting for the time‐varying CHODL association supported improved model fit (*p* = 0.019), indicating that the incremental genomic contribution was model‐dependent.

### 3.8. Immune Infiltration Landscape

As EBVaGC is characterized by a prominent immune‐active phenotype, we next examined immune infiltration patterns in both EBV‐related and tumor‐related cohorts. In the GEO_EBV cohort, EBV‐positive tumors showed significantly higher enrichment of multiple immune cell populations, including activated B cells, activated CD4 T cells, activated dendritic cells, macrophages, monocytes, and several memory T‐cell subsets, than EBV‐negative tumors (Figure S2A). Correlation analysis further showed that the five core genes were broadly positively associated with immune cell abundance, especially activated or memory T‐cell, B‐cell, and dendritic‐cell compartments (Figure S2B). In the TCGA–STAD cohort, tumor tissues also displayed marked remodeling of immune infiltration relative to normal tissues, and the core genes remained significantly correlated with diverse immune cell populations (Figures S3A and S3B). When patients were stratified by the median risk score, the high‐risk group exhibited increased enrichment of mast cells, activated dendritic cells, macrophages, monocytes, and several activated or memory T‐cell subsets (Figure S4A). Consistently, the risk score was positively correlated with most immune cell populations, with the strongest associations observed for mast cells, CD56bright NK cells, activated dendritic cells, macrophages, and effector or memory T‐cell subsets, whereas a negative correlation was observed for type 2 T helper cells (Figure S4B).

### 3.9. Single‐Cell Characterization and Cell Communication

**FIGURE 9:**
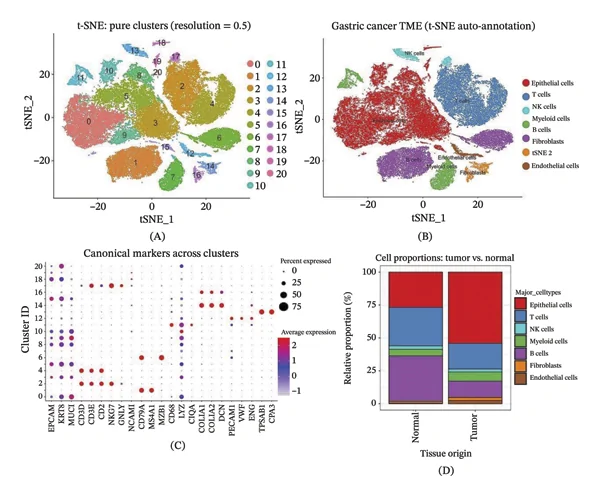
Single‐cell transcriptomic characterization of the gastric cancer tumor microenvironment based on GSE275648. (A) t‐SNE plot showing 21 transcriptionally distinct cell clusters identified after quality control, integration, and clustering. (B) t‐SNE plot showing annotation of the major cell lineages, including epithelial cells, T cells, NK cells, myeloid cells, B cells, fibroblasts, and endothelial cells. (C) Dot plot showing the expression of canonical marker genes across cell clusters for lineage annotation. (D) Stacked bar plot showing the relative proportions of major cell types in normal and tumor tissues.

To further contextualize the five‐gene signature at the cellular level, we analyzed the 11 directly deposited samples in GSE275648 (WCH_GC01–07 and WCH_NC01–04). After rigorous QC to remove doublets and low‐quality cells (Figures S5A and S5B) and subsequent dimensionality reduction, we identified 21 distinct cell clusters (Figure 9A). Based on the expression of canonical markers (Figure 9C), these clusters were annotated into seven major cell lineages: epithelial cells, T cells, NK cells, myeloid cells, B cells, fibroblasts, and endothelial cells (Figure 9B). Relative cell proportion analysis indicated a significant expansion of epithelial cells and T cells in tumor tissues compared to normal controls, reflecting the expansion of malignant clones and alterations in the immune landscape (Figure 9D).

Following cell type identification, we projected the expression of the five core biomarkers onto t‐SNE space to investigate their spatial distribution. The FeaturePlot visualization demonstrated highly cell‐type‐specific expression patterns (Figures S6A–S6E). Specifically, GNG7 and P2RY14 were predominantly localized in specific stromal cell clusters, while PI16 showed enriched expression within a subset of fibroblasts. Furthermore, the integrated five‐gene module score was also significantly enriched in fibroblasts and B cells compared to other lineages (Figure S6F). These findings suggest that the prognostic risk associated with these biomarkers is primarily driven by the stromal and specific immune components within the TME.

### 3.10. Altered Cellular Communication Networks in the Tumor Microenvironment

Given that both the five‐gene signature and the corresponding risk score were predominantly enriched in fibroblasts and B cells in the single‐cell analysis, we next investigated whether intercellular communication involving these cell populations was altered in the tumor microenvironment. CellChat analysis showed that the overall communication landscape was markedly remodeled in tumor tissue compared with normal tissue, with evident changes in the number and strength of inferred cell–cell interactions (Figures S7A and S7B). These findings indicate a global rewiring of intercellular signaling during tumor progression.

To further interpret this network reorganization in a biologically relevant context, we focused on fibroblasts and B cells, as these were the principal cell populations associated with the risk signature in the preceding analyses. Outgoing signaling analysis showed that fibroblasts occupied a prominent sender position in the tumor state and exhibited enhanced communication with multiple immune cell populations, particularly through MIF–(CD74 + CXCR4), MIF–(CD74 + CD44), and PTN–NCL signaling axes (Figure S7C). This pattern suggests that fibroblasts may actively participate in remodeling stromal–immune interactions within the tumor microenvironment.

In parallel, incoming signaling analysis identified B cells as an important recipient population in tumors. Specifically, TNFSF13B‐mediated signaling through TNFRSF17, TNFRSF13C, and TNFRSF13B, together with MIF–(CD74 + CXCR4) interactions, was markedly enriched in B cells in tumor tissue (Figure S8). These results suggest that B‐cell‐associated communication programs are selectively reinforced in the tumor microenvironment.

### 3.11. Prediction of Chemotherapeutic Sensitivity

**FIGURE 10:**
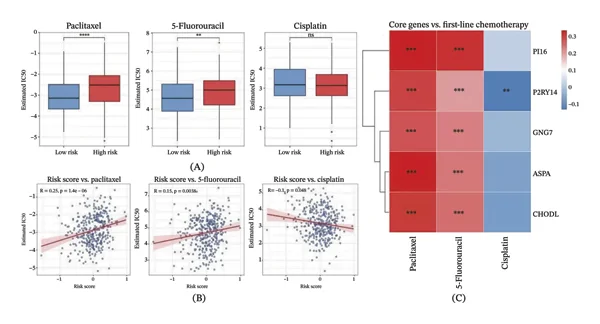
Predicted sensitivity to representative first‐line chemotherapeutic agents according to the risk model. (A) Box plots comparing the estimated IC50 values of paclitaxel, 5‐fluorouracil, and cisplatin between the high‐risk and low‐risk groups. (B) Scatter plots showing the correlations between risk score and estimated IC50 values for paclitaxel, 5‐fluorouracil, and cisplatin. (C) Heatmap showing the correlations between the five core biomarkers and the estimated sensitivity to representative first‐line chemotherapeutic agents.

A total of 86 compounds showed significant differences in predicted drug sensitivity between the high‐ and low‐risk groups. To enhance clinical relevance and interpretability, we focused on three representative agents commonly used in first‐line treatment of GC and presented their results in detail. Compared with the low‐risk group, high‐risk tumor samples showed significantly higher estimated IC50 values for paclitaxel and 5‐fluorouracil, indicating reduced predicted sensitivity to these agents, whereas no significant difference was observed for cisplatin (Figure 10A). Correlation analysis supported this pattern: The risk score was positively correlated with the estimated IC50 values of paclitaxel and 5‐fluorouracil and weakly negatively correlated with cisplatin (Figure 10B). At the gene level, all five core genes were positively associated with the estimated IC50 values of paclitaxel and 5‐fluorouracil, while correlations with cisplatin were weaker and generally inverse (Figure 10C).

## 4. Discussion

In this study, we integrated bulk transcriptomics, WGCNA, machine‐learning–based feature selection, immune deconvolution, and single‐cell analysis to identify a five‐gene signature associated with both EBV infection and gastric tumor status. The signature showed strong diagnostic performance across the discovery datasets and retained discriminatory capacity in independent datasets. By contrast, its stand‐alone prognostic performance was moderate and improved after integration with age and stage, indicating that this transcriptomic panel is better interpreted as a biologically informative stratifier rather than a fully sufficient prognostic tool by itself. Single‐cell mapping further localized the risk signal mainly to fibroblasts and B cells, suggesting that the clinical effect of the signature is closely linked to stromal–immune organization within the tumor microenvironment.

These findings are consistent with the current molecular framework of EBVaGC. The TCGA GC classification established EBV‐positive tumors as a distinct subtype characterized by recurrent PIK3CA alterations, extreme DNA hypermethylation, and amplification of immune‐related loci such as CD274 (PD‐L1) and PDCD1LG2 (PD‐L2) [15]. More recent reviews have emphasized that EBVaGC is not only molecularly distinct but also immunologically unique, showing coordinated viral, epigenetic, and immune regulatory remodeling [16]. In our study, the shared EBV‐ and tumor‐associated genes were enriched in cell‐cycle, cytokine‐cytokine receptor interaction, and PI3K–Akt pathways, whereas metabolic programs were suppressed. This pattern supports a model in which EBV‐associated gastric carcinogenesis emerges from the convergence of proliferative signaling, inflammatory activation, and metabolic rewiring rather than from a purely tumor‐cell‐intrinsic process.

The biological relevance of the five‐gene signature is supported by the known functions of its individual components. ASPA encodes aspartoacylase and has been linked to cell proliferation, invasion, and immune infiltration in some cancers, such as prostate cancer and hepatocellular carcinoma [17, 18], indicating that metabolic enzymes can participate in both tumor cell behavior and microenvironmental remodeling. GNG7, a γ‐subunit of heterotrimeric G proteins, is generally regarded as a tumor‐suppressive molecule in GC and has been reported to restrain cell‐cycle progression and promote apoptosis [19]. CHODL encodes a transmembrane adhesion‐related protein; although its role in GC remains insufficiently defined, dysregulated CHODL expression or methylation has been associated with tumor progression and survival in several solid tumors, suggesting a possible role in cell adhesion, migration, or niche adaptation [20, 21]. P2RY14 is a UDP‐sugar‐responsive purinergic receptor involved in inflammatory signaling and immune‐cell chemotaxis, and accumulating evidence indicates that its expression tracks immune and stromal programs within the tumor microenvironment [22]. PI16 is increasingly recognized as a marker of specialized fibroblast populations, and recent studies suggest that PI16‐positive fibroblasts can promote immune suppression and treatment resistance [23, 24]. Taken together, these functional features support the view that the present signature captures not only malignant‐cell alterations but also stromal and immune processes that are directly relevant to EBVaGC biology.

Our immune infiltration analyses help explain why an immune‐rich phenotype does not necessarily translate into a uniformly favorable outcome. In the GEO EBV cohort, EBV‐positive tumors showed higher enrichment of activated B cells, activated CD4 T cells, dendritic cells, macrophages, monocytes, and multiple memory T‐cell populations. However, the high‐risk group in TCGA was characterized not simply by increased immune infiltration but by enrichment of mast cells, macrophages, activated dendritic cells, CD56bright NK cells, and activated or memory T‐cell subsets. This pattern is compatible with the concept that inflamed tumors can still become functionally constrained or suppressive. Prior studies have likewise emphasized that EBVaGC often displays an immune‐hot phenotype, yet the composition, spatial organization, and activation state of infiltrating immune cells are critical determinants of outcome and immunotherapy response [25, 26]. In GC more broadly, B‐cell–rich tertiary lymphoid structures are often associated with improved immune surveillance and prognosis, though the state of B cells can differ substantially depending on the surrounding microenvironment [27]. Our results therefore suggest that the adverse phenotype captured by the five‐gene model reflects immune remodeling rather than simple immune abundance.

The single‐cell results further point to fibroblast‐centered signaling as a major component of this remodeling. Cancer‐associated fibroblasts are now recognized as central regulators of extracellular matrix architecture, cytokine gradients, immune cell trafficking, and therapeutic response, but their functions are highly heterogeneous and context‐dependent. In our data, the risk score was concentrated in fibroblasts and B cells, PI16 was enriched in a fibroblast subset, and fibroblasts acted as dominant signal senders in tumor tissue. These observations are biologically plausible because PI16‐positive fibroblasts have been described as a distinct fibroblast state with immunomodulatory potential [28, 29], and fibroblast‐derived PI16 has recently been linked to Treg induction and poor outcome in squamous cancer [24]. In parallel, our CellChat analysis highlighted enhanced MIF–(CD74 + CXCR4), MIF–(CD74 + CD44), PTN–NCL, and TNFSF13B‐related signaling in the tumor state. This is notable because MIF—CD74–CXCR4/CD44 signaling has been implicated in immunosuppressive barrier formation and therapy resistance in GC [30]. Together, these data support the idea that the prognostic impact of the signature arises largely from fibroblast‐immune crosstalk rather than from epithelial expression alone.

A testable explanation for the apparently paradoxical coexistence of immune enrichment and adverse outcome is that immune abundance and immune effectiveness are not equivalent. In the single‐cell dataset, five‐gene UCell activity was concentrated in fibroblast and B‐cell compartments, while CellChat predicted enhanced fibroblast‐associated MIF–CD74/CXCR4 or MIF–CD74/CD44 communication and reinforced TNFSF13B receptor signaling into B cells. We therefore propose, but do not prove, that a PI16‐associated fibroblast state may help organize an inflamed yet functionally constrained niche: MIF‐related signaling could alter immune‐cell recruitment or state, whereas concurrent BAFF‐family inputs could sustain selected B‐cell populations without guaranteeing effective antitumor immunity. In this framework, the bulk prognostic score may partly reflect the abundance or activation state of these stromal–immune compartments rather than a purely epithelial program. Because donor EBV status was unavailable and CellChat provides computational inference rather than direct interaction measurements, this model should be regarded as a gastric‐cancer microenvironment hypothesis requiring spatial and functional validation, not as an established EBVaGC‐specific mechanism.

The drug‐sensitivity analysis should also be interpreted in this microenvironmental context. High‐risk tumors had higher predicted IC50 values for paclitaxel and 5‐fluorouracil, suggesting reduced sensitivity rather than enhanced vulnerability to these first‐line agents, while cisplatin‐related differences were limited. This finding is directionally consistent with the view that stromal remodeling and immune suppression can accompany drug resistance [31]. Clinically, this suggests that the five‐gene signature may have greater value for identifying biologically aggressive, microenvironment‐dominated tumors than for nominating immediate chemotherapy benefits. Given the known association of EBVaGC with immune checkpoint biology, future work should test whether this signature can refine patient stratification for chemotherapy, immunotherapy, or combined treatment in cohorts with detailed response data.

Several limitations should be acknowledged. First, the machine learning–based gene selection was performed without an explicit training/test split, which may introduce a risk of overfitting. Future studies should incorporate independent training and validation cohorts to further confirm the robustness of the selected gene signature. Second, although the diagnostic signal was strong, the prognostic model alone showed only moderate discrimination, underscoring the need for prospective validation and model recalibration in clinically annotated cohorts. Third, functional experiments will be required to determine whether PI16‐, P2RY14‐, or GNG7‐related pathways directly shape fibroblast‐immune communication in EBVaGC. In addition, protein‐level validation was not performed in this study. Although PI16 showed fibroblast‐enriched expression at the single‐cell transcriptomic level, immunohistochemical validation in EBV‐stratified tissue microarrays is required to confirm its translational relevance. Finally, the scRNA‐seq dataset used in this study was obtained from the public GSE275648 cohort, and EBV status was not available for individual donors in the deposited metadata. Therefore, although the single‐cell and CellChat analyses helped localize the five‐gene signature to fibroblast‐ and B‐cell‐associated compartments and provided useful information on the GC tumor microenvironment, these findings should not be regarded as direct EBVaGC‐specific single‐cell validation. Future studies using EBV‐status‐defined single‐cell cohorts or EBV‐stratified patient tissues are needed to confirm whether the fibroblast‐immune communication patterns observed here are specifically related to EBVaGC.

In conclusion, we identified a five‐gene transcriptomic signature associated with complementary EBV‐related and gastric tumor‐related features that showed robust diagnostic performance across cohorts and provided biologically interpretable prognostic stratification. Integrative bulk and single‐cell analyses further suggested that the adverse risk phenotype is linked to fibroblast‐ and B‐cell‐associated microenvironmental remodeling and altered intercellular communication, supporting future biomarker studies in EBVaGC.

## Author Contributions

Lufei Jin: writing–original draft, software, data curation, conceptualization. Man Jiang: writing–original draft, resources, methodology. Yubin Pan: validation, software, visualization. Yan Wang: validation, methodology. Lei Zhang: validation, supervision, investigation. Wujie Chen: writing–review and editing, supervision, investigation, conceptualization.

## Funding

This work was supported by the Medical and Health Science Program of Zhejiang Province (grant no. 2025HY0241), the Zhejiang Provincial Traditional Chinese Medicine Science and Technology Project (grant no. 2024ZL314), and the National Natural Science Foundation Cultivation Project of Zhejiang Cancer Hospital (grant no. PY2024041).

## Ethics Statement

The authors have nothing to report.

## Consent

All authors have read and approved the final version of the manuscript and consent to its submission for publication.

## Conflicts of Interest

The authors declare no conflicts of interest.

## Supporting Information

Additional supporting information can be found online in the Supporting Information section.

## Supporting information


**Supporting Information 1** Figure S1. Intersection of common DEGs with EBV‐ and tumor‐associated WGCNA modules.

**Supporting Information 2** Figure S2. Immune infiltration landscape associated with the five core genes in the GEO EBV cohort. (A) Comparison of immune infiltration scores between EBV‐positive and EBV‐negative samples in the GSE51575 cohort. (B) Heatmap showing the correlations between expression of the five core genes and the abundance of infiltrating immune cell populations in the GEO EBV cohort.

**Supporting Information 3** Figure S3. Immune infiltration landscape associated with the five core genes in the TCGA–STAD cohort. (A) Comparison of immune infiltration scores between tumor and normal samples in the TCGA–STAD cohort. (B) Heatmap showing the correlations between expression of the five core genes and the abundance of infiltrating immune cell populations in TCGA–STAD.

**Supporting Information 4** Figure S4. Risk‐score–associated immune infiltration patterns. (A) Comparison of immune infiltration scores between the high‐risk and low‐risk groups defined by the prognostic model. (B) Lollipop plot showing the correlations between risk score and immune cell abundance across the evaluated immune cell populations.

**Supporting Information 5** Figure S5. Quality control and feature‐selection workflow for single‐cell RNA‐seq analysis. (A) Violin plots showing post‐QC distributions of nFeature_RNA and nCount_RNA across individual samples after removal of doublets and low‐quality cells. (B) Violin plots showing post‐QC distributions of nFeature_RNA and nCount_RNA according to tissue origin (normal vs. tumor). (C) Identification of highly variable genes used for downstream dimensionality reduction and clustering. (D) Elbow plot showing principal component selection for downstream single‐cell analysis.

**Supporting Information 6** Figure S6. Cell‐type‐specific expression of the five core biomarkers and distribution of the risk signature score. (A‐E) Feature plots showing the expression patterns of ASPA, CHODL, GNG7, P2RY14, and PI16 across the single‐cell t‐SNE map. (F) Feature plots showing the distribution of the integrated risk signature score across the single‐cell t‐SNE map.

**Supporting Information 7** Figure S7. Fibroblast‐centered communication remodeling in the tumor microenvironment. (A) Global cell–cell communication network showing the overall interaction structure among major cell populations. (B) Scatter plot of outgoing and incoming signaling strengths comparing signaling roles of each cell lineage in normal and tumor tissues. (C) Bubble plot showing ligand–receptor pairs mediating fibroblast‐centered outgoing signaling toward T cells, NK cells, and myeloid cells in normal and tumor tissues.

**Supporting Information 8** Figure S8. B‐cell–associated communication programs in the tumor microenvironment. (A) Bubble plot showing major outgoing ligand–receptor signaling pathways originating from B cells in normal and tumor tissues. (B) Bubble plot showing major incoming ligand–receptor signaling pathways targeting B cells in normal and tumor tissues.

**Supporting Information 9** Table S1 Primer sequences used for quantitative real‐time PCR analysis.

**Supporting Information 10** Table S2 Benchmarking of the five‐gene prognostic model against clinical models in the TCGA–STAD cohort.

## Data Availability

TCGA–STAD transcriptomic and clinical data are available through the NCI Genomic Data Commons. GSE51575 and GSE27342 are available through the Gene Expression Omnibus. The public single‐cell data were obtained from GEO Series GSE275648; the directly deposited WCH samples are GSM8481907–GSM8481917. No new bulk or single‐cell omics data were generated in the present study.
