# Supplementary material for: Integrated Bulk and Single‐Cell Transcriptomic Analyses Identify a Five‐Gene Signature Associated With Prognosis and the Tumor Microenvironment in Epstein–Barr Virus–Associated Gastric Cancer
Source: Genet Res (Camb). 2026 Aug 29;2026:5991107. doi: 10.1155/genr/5991107 (PMC13525711; doi:10.1155/genr/5991107)
Supplement: Supplementary file 1 [file GENR-2026-5991107-s007.docx]

Table S1 Primer sequences used for quantitative real-time PCR analysis

| Primer Name | | Sequence (5'->3') |
| --- | --- | --- |
| ASPA | Forward primer | TGCCCTTTGGGTAAAGTCTCA |
|  | Reverse primer | ATAGACCTCAATGGCGCAGG |
| CHODL | Forward primer | TTGGGATTGACACTGGAGGC |
|  | Reverse primer | TCTCAAGCAAACCCTCATCAA |
| GNG7 | Forward primer | TTGGTTTCGGGATCTCGGTG |
|  | Reverse primer | CGCTCAATCCCGGCTTCTAT |
| P2RY14 | Forward primer | GGAAAAATTGGCCTCTGCCT |
|  | Reverse primer | ATTTCCCTAAACGGCTGGCA |
| PI16 | Forward primer | CCAGGCCCCAACTTCCTTAG |
|  | Reverse primer | ACCTCTTCACCCTTTGAGTGG |
| GAPDH | Forward primer | GGGTCCCAGCTTAGGTTCAT |
|  | Reverse primer | TGAGGTCAATGAAGGGGTCG |
