# Supplementary material for: Integrated Bulk and Single‐Cell Transcriptomic Analyses Identify a Five‐Gene Signature Associated With Prognosis and the Tumor Microenvironment in Epstein–Barr Virus–Associated Gastric Cancer
Source: Genet Res (Camb). 2026 Aug 29;2026:5991107. doi: 10.1155/genr/5991107 (PMC13525711; doi:10.1155/genr/5991107)
Supplement: Supplementary file 2 [file GENR-2026-5991107-s008.docx]

Table S2 Benchmarking of the five-gene prognostic model against clinical models in the TCGA-STAD cohort

| Model | C-index | Corrected C | AUC 12 m | AUC 24 m | AUC 36 m |
| --- | --- | --- | --- | --- | --- |
| TNM stage | 0.628(0.580-0.685) | 0.621 | 0.636 | 0.643 | 0.587 |
| Age + TNM stage | 0.663(0.613-0.723) | 0.652 | 0.671 | 0.719 | 0.654 |
| Five-gene component | 0.607(0.560-0.678) | 0.578 | 0.648 | 0.641 | 0.611 |
| Age + TNM + five genes | 0.694(0.655-0.757) | 0.665 | 0.713 | 0.769 | 0.693 |
